# Supplementary material for: Mechanistic models of PLC/PKC signaling implicate phosphatidic acid as a key amplifier of chemotactic gradient sensing
Source: PLoS Comput Biol. 2020 Apr 7;16(4):e1007708. doi: 10.1371/journal.pcbi.1007708 (PMC7164671; doi:10.1371/journal.pcbi.1007708)
Supplement: S1 Text — (PDF) [file pcbi.1007708.s001.pdf]

SUPPLEMENTARY TEXT S1: Modeling details

"Mechanistic Models of PLC/PKC Signaling Implicate Phosphatidic Acid as a Key Amplifier of Chemotactic Gradient Sensing," by Nosbisch et al.

General forms of the differential equations and boundary conditions

Species (**Table S1**) reside in either the cytosol or the plasma membrane. For each cytosolic species *i*, we can write a general conservation equation as follows.

$$\frac{\partial C_i}{\partial t} = D_i \nabla^2 C_i + R_{V,i}$$

Here, C_i is the local concentration of species *i* (µM), *t* is time (s), D_i is the molecular diffusivity of species *i* (µm^2/s), and $R_{V,i}$ is the volumetric production rate of species *i* (µM/s). The associated boundary condition at the plasma membrane surface, *S*, equates the diffusive flux to the net rate of production at the membrane, $R_{S,i}$ (µm^{-2} s^{-1}).

$$D_i(\mathbf{n} \cdot \nabla C_i)|_S = (0.00166\ \mu\text{M}\ \mu\text{m}^3) R_{S,i}$$

The constant on the right-hand side incorporates Avogadro's number and the conversion of volume units from L to µm^3, which is done automatically in Virtual Cell.

For each membrane species *i*, conservation equations have the following form:

$$\frac{\partial c_i}{\partial t} = D_i \nabla^2 c_i + r_i$$

Here, c_i is the local area density of membrane species *i* (#/µm^2), D_i is the molecular diffusivity of membrane species *i* (µm^2/s), and r_i is the production rate of membrane species *i*.

Molecular diffusivities and initial concentrations

The assigned diffusivity of receptor-bound PLC is 0.01 µm^2/s, which is typical of transmembrane receptors and effectively immobile. We assigned diffusivities for PIP_2, DAG, PA, DAG-PKC, and DAG-PKC* equal to 0.5 µm^2/s, a value typical for plasma membrane lipids. Diffusivities of cytosolic species vary modestly according to hydrodynamic radius, scaling approximately with molecular weight (MW) as $D \propto MW^{-1/3}$ and are of the order of magnitude estimated from photobleaching measurements in cytoplasm. These diffusivity values are the same as those assumed in Mohan et al. [1]. Initial conditions are also the same as in Mohan et al., except for the initial concentration of inactive PLC enzyme, which was modestly reduced from 0.03 to 0.02 µM. As explained previously [1], the initial abundance of membrane-bound MARCKS, together

with its affinity for PIP_2, was chosen such that 90% of the PIP_2 molecules and 90% of the MARCKS molecules in the cell are initially in complex.

Table S1: List of model species, their diffusivities, and their initial concentrations

Variable	Description	Compartment	Diffusivity (μm²/s)	Initial Condition
E	Inactive PLC enzyme	Cytosol	19	0.02 μM
M	Unphosphorylated MARCKS	Cytosol	32	1 μM
M_p	Phosphorylated MARCKS	Cytosol	32	0
C	Inactive PKC	Cytosol	24	0.3 μM
e	Active PLC enzyme	Membrane	0.01	0
p_T	Total PIP_2	Membrane	0.5	5000 μm^{-2}
m_T	Total membrane MARCKS	Membrane	0.5	3727 μm^{-2}
d	Free DAG	Membrane	0.5	0
d_p	PA	Membrane	0.5	0
c^*	Active DAG-PKC	Membrane	0.5	0
c	Inactive DAG-PKC	Membrane	0.5	0

Production rates

Here, we define the aforementioned production rates for the cytosolic species ($R_{V,i}$ and $R_{S,i}$) and membrane species (r_i) in terms of the net rates of reactions, V_j. Rate law expressions for the V_j's are covered in the next section.

Inactive PLC enzyme (E): Recruited to the membrane with net flux V_{PLC}.

$$R_{V,E} = 0;\ \ R_{S,E} = -V_{PLC}$$

Unphosphorylated MARCKS (M): Generated by dephosphorylation at rate V_{dpM}; recruited to the membrane with net flux V_{MARCKS}.

$$R_{V,M} = V_{dpM};\ \ R_{S,M} = -V_{MARCKS}$$

Phosphorylated MARCKS (M_p): Consumed by dephosphorylation at rate V_{dpM}; generated by phosphorylation of membrane-associated MARCKS, with flux V_{pm}.

$$R_{V,Mp} = -V_{dpM};\ \ R_{S,Mp} = V_{pm}$$

Inactive PKC (C): Binds DAG in an active form with net flux V_{PKC}; recovered by release of inactive DAG-PKC with flux $V_{off,c}$.

$$R_{V,C} = 0;\ \ R_{S,C} = -V_{PKC} + V_{off,c}$$

Active PLC enzyme (e): Recruited with net flux V_{PLC}.

$$r_e = V_{PLC}$$

Total PIP_2 (p_T): Synthesized at a constant rate, $V_{synth,p}$; consumed with basal and active PLC-catalyzed rates ($V_{basal,p}$ and $V_{hyd,PLC}$, respectively).

$$r_{p_T} = V_{synth,p} - V_{basal,p} - V_{hyd,PLC}$$

Total membrane MARCKS (m_T): Recruited to the membrane with the net flux V_{MARCKS}; released from the membrane through phosphorylation by PKC with flux V_{pm}.

$$r_{m_T} = V_{MARCKS} - V_{pm}$$

Free DAG (d): Produced from PLC-catalyzed hydrolysis of PIP_2 with rate $V_{hyd,PLC}$; consumed via diacylglycerol kinase (DAGK)-mediated phosphorylation with rate V_{DAGK}; consumed by net binding to PKC with rate V_{PKC}; recovered by release of inactive DAG-PKC with rate $V_{off,c}$. In addition, the present models include generation of DAG from dephosphorylation of PA by PA phosphatase, with rate V_{PAP}.

$$r_d = V_{hyd,PLC} - V_{DAGK} - V_{PKC} + V_{off,c} + V_{PAP}$$

PA (d_p): Produced via DAGK-catalyzed phosphorylation with rate V_{DAGK} and by phospholipase D (PLD)-catalyzed hydrolysis of phosphatidylcholine with rate V_{PLD}; consumed with basal rate $V_{basal,dp}$ and conversion of PA to DAG by PA phosphatase with rate V_{PAP}.

$$r_{d_p} = V_{DAGK} + V_{PLD} - V_{basal,dp} - V_{PAP}$$

Active DAG-PKC (c^*): Recruited from the cytosol with net rate, V_{PKC}; deactivated via dephosphorylation with rate V_{dpc}; recovered via re-phosphorylation of the inactive form with rate V_{pc}.

$$r_{c^*} = V_{PKC} - V_{dpc} + V_{pc}$$

Inactive DAG-PKC (c): Generated via dephosphorylation of the active form with rate V_{dpc}; consumed by phosphorylation with rate V_{pc}; dissociates from DAG with rate $V_{off,c}$.

$$r_c = V_{dpc} - V_{pc} - V_{off,c}$$

Rate law expressions

All rate laws are listed in **Table S2**. As explained in Mohan et al. [1], most of them are based on simplified kinetic principles (mass-action binding, pseudo-first-order and pseudo-second-order reactions). For V_{MARCKS} (binding of unphosphorylated MARCKS to the membrane), we assume that MARCKS initially associates with the membrane via reversible insertion of its myristoyl lipid, and is stabilized by quasi-equilibrium binding to PIP_2; the polybasic motif of MARCKS is modeled as three equivalent binding sites for PIP_2. As previously derived [1], these assumptions allow one to calculate p, the density of free (unbound) PIP_2 from p_T, m_T, and the single-site equilibrium constant of MARCKS-PIP_2 binding, K_{PIP2}.

$$p = \frac{K_{PIP2}p_T - 3K_{PIP2}m_T - 1 + [(K_{PIP2}p_T - 3K_{PIP2}m_T - 1)^2 + 4K_{PIP2}p_T]^{1/2}}{2K_{PIP2}}$$

The rate laws involving p, including V_{MARCKS}, are given in Table S2.

The net rate of PLC binding to the membrane, V_{PLC}, is also given in Eqn. 2 of the main text and has been modified in the following ways relative to the model presented in Mohan et al. In the association rate, active receptor density, r, is taken as a model input (Eqn. 1 in the main text), and we account for the depletion of free PLC binding sites (density $r - e$). The dissociation rate accounts for positive feedback loop (PFL) 1 and considers that receptor-bound PLC is in pseudo-equilibrium with the local concentration of PA (d_p). Taking K_{PA} as the associated equilibrium constant, the density of membrane-bound PLC that is bound to PA according to this approximation is $\left(\frac{K_{PA}d_p}{1+K_{PA}d_p}\right)e$, while the density of membrane-bound PLC that is not bound to PA is $\left(\frac{1}{1+K_{PA}d_p}\right)e$. While bound to PA, if PLC dissociates from the active receptor complex, it will either dissociate from PA and join the cytosolic pool or rebind the receptor (by diffusion-controlled capture) before that happens. Taking ε as a (presumably low) constant probability that PLC will dissociate from PA before recapture, we obtained the rate expression given in Table S2 and in Eqn. 2 of the main text.

The rate of PA production by PLD, V_{PLD}, is a new reaction in the present models. It introduces a second route of PA generation and reflects the influence of PFL 2. A Hill function is assumed, as explained in the main text (Eqn. 3).

Table S2: Reaction rate expressions

Rate	Description	Expression	Base parameters
V_{PLC}	Net PLC binding with enhancement by PA (PFL 1)	$k_{on,e}(r-e)E\|_S - k_{off,e}\left(\frac{1+\varepsilon K_{PA}d_p}{1+K_{PA}d_p}\right)e$	$k_{on,e}$: 0.1 μM^{-1}s^{-1}<br>$k_{off,e}$: 0.1 s^{-1}<br>ε: 0.01<br>K_{PA}: 10 μm^2
$V_{synth,p}$	Production of free PIP_2	$V_{synth,p}$	$V_{synth,p}$: 2.5 μm^{-2}s^{-1}
$V_{basal,p}$	Basal PIP_2 consumption	$k_{basal,p}p$	$k_{basal,p}$: 0.005 s^{-1}
$V_{hyd,PLC}$	PIP_2 hydrolysis by PLC	$k_{hyd,PLC}e\,p$	$k_{hyd,PLC}$: 0.0005 μm^{2}s^{-1}
V_{MARCKS}	Net MARCKS binding to PIP_2	$k_{on,m}M\|_S - \frac{k_{off,m}m}{(1+K_{PIP2}p)^3}$	$k_{on,m}$: 792 μM^{-1}μm^{-2}s^{-1}<br>$k_{off,m}$: 1 s^{-1}<br>K_{PIP2}: 0.00135 μm^2
V_{pm}	MARCKS phosphorylation	$k_{pm}c^*m$	k_{pm}: 1 μm^{2}s^{-1}
V_{dpm}	MARCKS dephosphorylation	$k_{dpM}M_p$	k_{dpM}: 0.3 s^{-1}
$V_{basal,dp}$	Basal PA consumption	$k_{basal,dp}d_p$	$k_{basal,dp}$: 1 s^{-1}
V_{DAGK}	DAG conversion to PA	$k_{DAGK}d$	k_{DAGK}: 1 s^{-1}
V_{PAP}	PA conversion to DAG	$k_{PAP}d_p$	k_{PAP}: 1 s^{-1}
V_{PKC}	Net PKC binding to DAG	$k_{on,c}d\,C\|_S - k_{off,c}c^*$	$k_{on,c}$: 1 μM^{-1}s^{-1}<br>$k_{off,c}$: 0.1 s^{-1}
V_{dpc}	Dephosphorylation of PKC	$k_{dpc}c^*$	k_{dpc}: 1 s^{-1}
V_{pc}	PKC re-phosphorylation	$k_{pc}c$	k_{pc}: 1 s^{-1}
$V_{off,c}$	Dissociation of inactive PKC	$k_{off,c}c$	$k_{off,c}$: 0.1 s^{-1}
V_{PLD}	Production of PA with enhancement by active PKC (PFL 2)	$V_{synth,dp}\left(\frac{1+\gamma(K_{PLD}c^*)^n}{1+(K_{PLD}c^*)^n}\right)$	$V_{synth,dp}$: 0.0001 μm^{-2}s^{-1}<br>γ: variable<br>K_{PLD}: variable<br>n: 2

Specification of rate parameters

Where reasonable, we kept values of rate constants the same as in the previous modeling study [1]. Exceptions and new rate parameters are discussed below.

In the Mohan model, parameters $k_{on,e}$ and $k_{off,e}$ in V_{PLC} were chosen to yield fast kinetics and a dissociation constant ($k_{off,e}/k_{on,e}$) of 10 nM. To achieve that, the previous value of $k_{on,e}$ (10 $\mu M^{-1}s^{-1}$, or 10^7 $M^{-1}s^{-1}$) was at the high end of the observed range for protein-protein interactions. Furthermore, those kinetics did not account for enhancement of PLC recruitment by PA. Therefore, we reduced the value of $k_{on,e}$ by two logs, to 0.1 $\mu M^{-1}s^{-1}$ (10^5 $M^{-1}s^{-1}$), well within the observed range for protein-protein interactions, to offset the effect of PA (characterized by the chosen value of the escape probability, $\varepsilon = 0.01$). The other, new parameter associated with V_{PLC} is the PLC-PA equilibrium constant, K_{PA}. For the base case, it was set to an order-of-magnitude value of 10 μm^2, since the maximum values of d and d_p are ~ 1 μm^{-2}.

The new way we are handling receptor activation in these models results in a substantially higher magnitude of the active receptor density; even though the characteristic receptor density is the same, the receptor dynamics considered in Mohan et al. resulted in a small fraction of receptors activated, even at saturating [PDGF]. To adjust for that, such that a comparable rate of PIP_2 hydrolysis was achieved, we reduced the initial PLC concentration modestly (0.02 μM, from 0.03 μM) and the value of $k_{hyd,PLC}$ by a factor of 10 (0.0005 $\mu m^2 s^{-1}$, from 0.005 $\mu m^2 s^{-1}$). The other parameter that was adjusted, modestly, is the pseudo-first-order rate constant characterizing MARCKS dephosphorylation in the cytosol (0.3 s^{-1}, reduced from 1 s^{-1}). Collectively these changes bring the effects of MARCKS regulation on the PKC pathway in line with those characterized in Mohan et al. [1].

In the modeling by Mohan et al., PA was an implicit variable, as the phosphorylation of DAG by DAGKs was considered irreversible. As stated above, the present models consider the reverse reaction, with rate V_{PAP} and pseudo-first-order rate constant k_{PAP}. As a base case, we chose $k_{PAP} = k_{DAGK} = 1$ s^{-1}. The effect of this parameter on the relative densities of DAG and PA are elucidated in the following section. PA is also generated by a newly considered reaction: hydrolysis of phosphatidylcholine catalyzed by PLD. In the associated rate expression, V_{PLD}, the phosphatidylcholine concentration does not appear, as it is assumed relatively abundant and approximately constant. To simplify the analysis, we chose an extremely low value of the basal synthesis rate ($V_{synth,dp}$), such that the effect of this reaction is negligible in the absence of PFL

2. In effect, the rate constant for PLD is the product, $V_{synth,dp}\gamma$, and that is why it is stated that way in the caption of **Fig. 5**. As explained in the main text, extensive parameter sweeps of K_{PLD} and γ were run to determine optimal values for amplification by PFL 2 in conjunction with MARCKS, PFL 1, or both MARCKS and PFL 1.

Steady state analysis of DAG and PA levels (no PFL 2)

A steady-state analysis, assuming negligible spatial gradients of the lipid species, predicts a consistent proportional relationship between the concentrations of DAG and PA, dependent on just four parameters (k_{DAGK}, $k_{basal,dp}$, k_{PAP}, and $V_{synth,dp}$). The derivation for this relationship follows, starting with the reaction-diffusion equation for PA:

$$\frac{\partial d_p}{\partial t} = D_i \nabla^2 d_p + r_{d_p}$$

Assuming a steady state has been reached and neglecting the diffusion term,

$$\frac{\partial d_p}{\partial t} = 0 \approx r_{d_p} = V_{DAGK} + V_{PLD} - V_{basal,dp} - V_{PAP}$$

Substituting in the rate expressions, with no PFL 2 ($V_{PLD} = V_{synth,dp}$) and rearranging,

$$k_{DAGK} d + V_{synth,dp} - k_{basal,dp} d_p - k_{PAP} d_p \approx 0$$

$$k_{DAGK} d + V_{synth,dp} \approx \left(k_{basal,dp} + k_{PAP}\right) d_p$$

$$d_p \approx \left(\frac{k_{DAGK}}{k_{basal,dp} + k_{PAP}}\right) d + \frac{V_{synth,dp}}{k_{basal,dp} + k_{PAP}}$$

For our set of parameters, the second term is negligible, and therefore

$$d_p \approx \left(\frac{k_{DAGK}}{k_{basal,dp} + k_{PAP}}\right) d$$

Another, related analysis was to plot *e*- and 'DAG'-nullclines with free DAG (*d*) on the abscissa and active PLC (*e*) on the ordinate. For the *e*-nullcline,

$$r_e = V_{PLC} = k_{on,e}(r - e)E|_S - k_{off,e}\left(\frac{1 + \varepsilon K_{PA} d_p}{1 + K_{PA} d_p}\right) e = 0;$$

$$e = \frac{k_{on,e} r E|_S}{k_{on,e} E|_S + k_{off,e}\left(\frac{1 + \varepsilon K_{PA} d_p}{1 + K_{PA} d_p}\right)},$$

and the linear relationship between d_p and d derived above, in the absence of PFL 2, is invoked. The concentration of PLC in the cytosol, E, was approximately uniform at steady state, and its value was obtained from the corresponding simulation. The e-nullcline equation also includes the local density of active receptors, r. For the 'DAG'-nullcline, we consider both free and bound forms of DAG:

$$r_d + r_{c^*} + r_c = V_{hyd,PLC} - V_{DAGK} + V_{PAP} = k_{hyd,PLC}\, e\, p - k_{DAGK} d + k_{PAP} d_p = 0.$$

Incorporating the approximately proportional relationship between d_p and d derived above, the DAG-nullcline is, approximately,

$$e = \left(\frac{k_{basal,dp}}{k_{basal,dp} + k_{PAP}}\right) \frac{k_{DAGK}}{k_{hyd,PLC}\, p} d.$$

The local densities of free PIP_2, p, at the front and back of the cell were obtained from the corresponding simulation.

References

1. Mohan K, Nosbisch JL, Elston TC, Bear JE, Haugh JM. A reaction-diffusion model explains amplification of the PLC/PKC pathway in fibroblast chemotaxis. Biophysical Journal. 2017; 113: 185-94.
