# Supplementary figures and images for: Mechanistic models of PLC/PKC signaling implicate phosphatidic acid as a key amplifier of chemotactic gradient sensing
Source: PLoS Comput Biol. 2020 Apr 7;16(4):e1007708. doi: 10.1371/journal.pcbi.1007708 (PMC7164671; doi:10.1371/journal.pcbi.1007708)

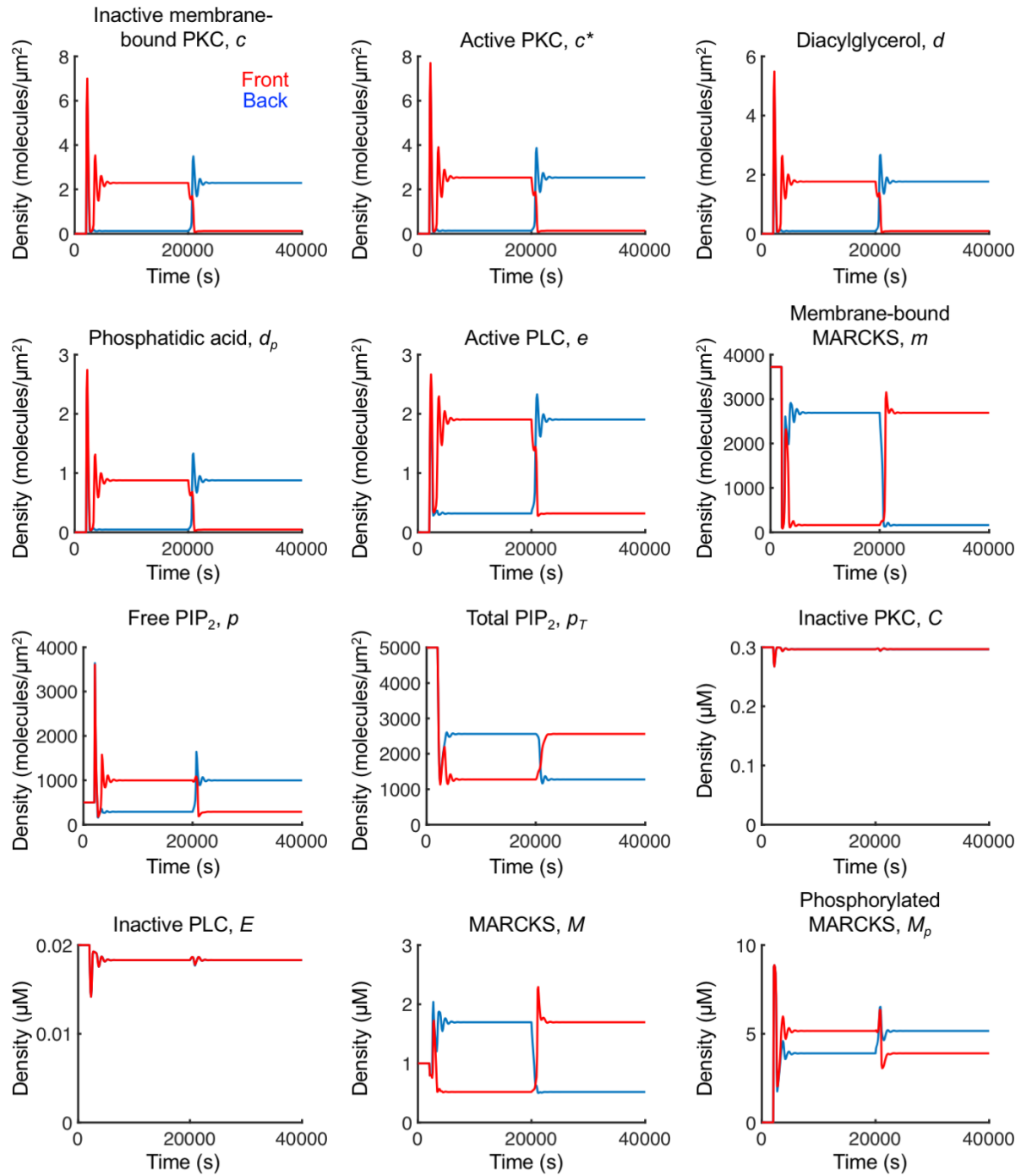

**S1 Fig: Time courses of all model species for Fig. 2A, 10% steepness and  $rfrac = 0.1$ .**

Supplement: S1 Fig — (PDF) [file pcbi.1007708.s003.pdf]

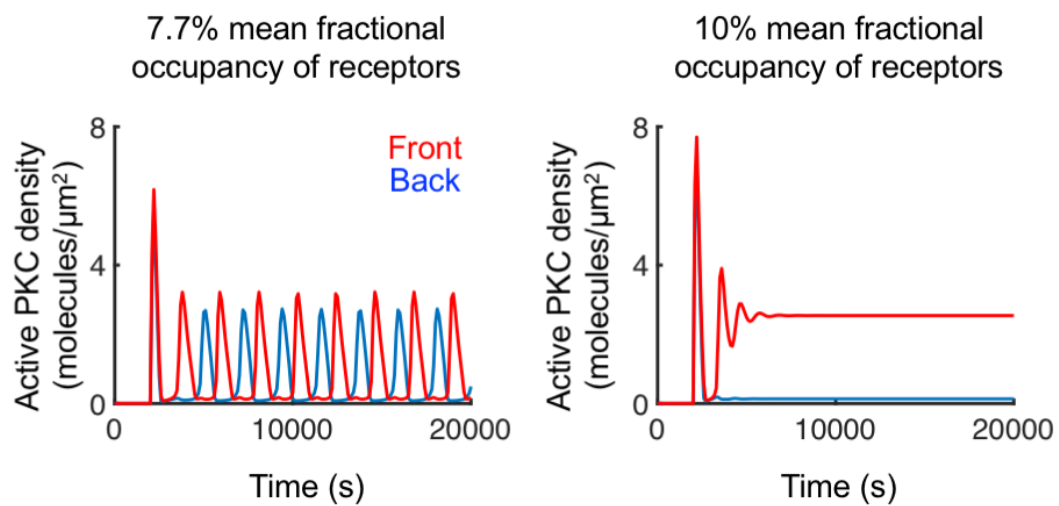

**S2 Fig: Transition from oscillations to stable pattern as *rfrac* is increased (10% steepness).**

Supplement: S2 Fig — (PDF) [file pcbi.1007708.s004.pdf]

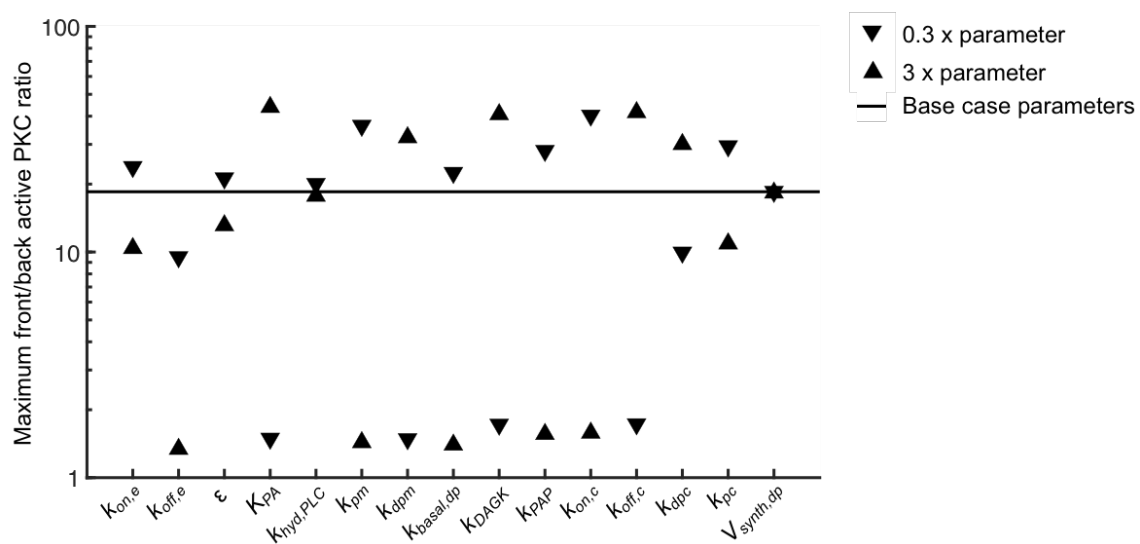

**S4 Fig: Systematic variation of kinetic parameters (no PFL 2).**

Supplement: S4 Fig — (PDF) [file pcbi.1007708.s006.pdf]
