# Supplementary material for: Mechanistic models of PLC/PKC signaling implicate phosphatidic acid as a key amplifier of chemotactic gradient sensing
Source: PLoS Comput Biol. 2020 Apr 7;16(4):e1007708. doi: 10.1371/journal.pcbi.1007708 (PMC7164671; doi:10.1371/journal.pcbi.1007708)
Supplement: S3 Fig — These correspond to the simulations analyzed in Fig 2A with 10% gradient steepness. The plot with rfrac = 0.1 is the same as in Fig 3A. When rfrac = 0.0278, DAG is low at both the front and back of the cell; with rfrac = 0.278, DAG is high at both the front and back of the cell. (PDF) [file pcbi.1007708.s005.pdf]

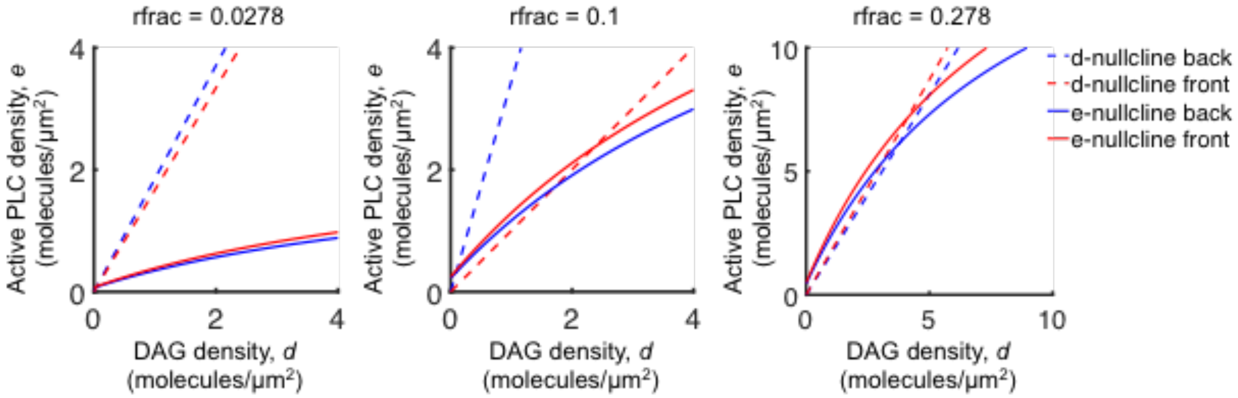


**S3 Fig: Phase plots for different *rfrac* values.** These correspond to the simulations analyzed in **Fig. 2A** with 10% gradient steepness. The plot with *rfrac* = 0.1 is the same as in **Fig. 3A**. When *rfrac* = 0.0278, DAG is low at both the front and back of the cell; with *rfrac* = 0.278, DAG is high at both the front and back of the cell.
