# Supplementary material for: Mechanistic models of PLC/PKC signaling implicate phosphatidic acid as a key amplifier of chemotactic gradient sensing
Source: PLoS Comput Biol. 2020 Apr 7;16(4):e1007708. doi: 10.1371/journal.pcbi.1007708 (PMC7164671; doi:10.1371/journal.pcbi.1007708)
Supplement: S5 Fig — The parameters targeted were kDAGK, which affects the conversion of DAG to PA; KPA, which affects the PA-PLC affinity in PFL 1; and KPLD, which affects the sensitivity of PFL 2 to active PKC. Each parameter was decreased to 0.3X and increased to 3X. Each plot shows active PKC density vs. rfrac for 10% gradient steepness, as in Fig 6. (PDF) [file pcbi.1007708.s007.pdf]

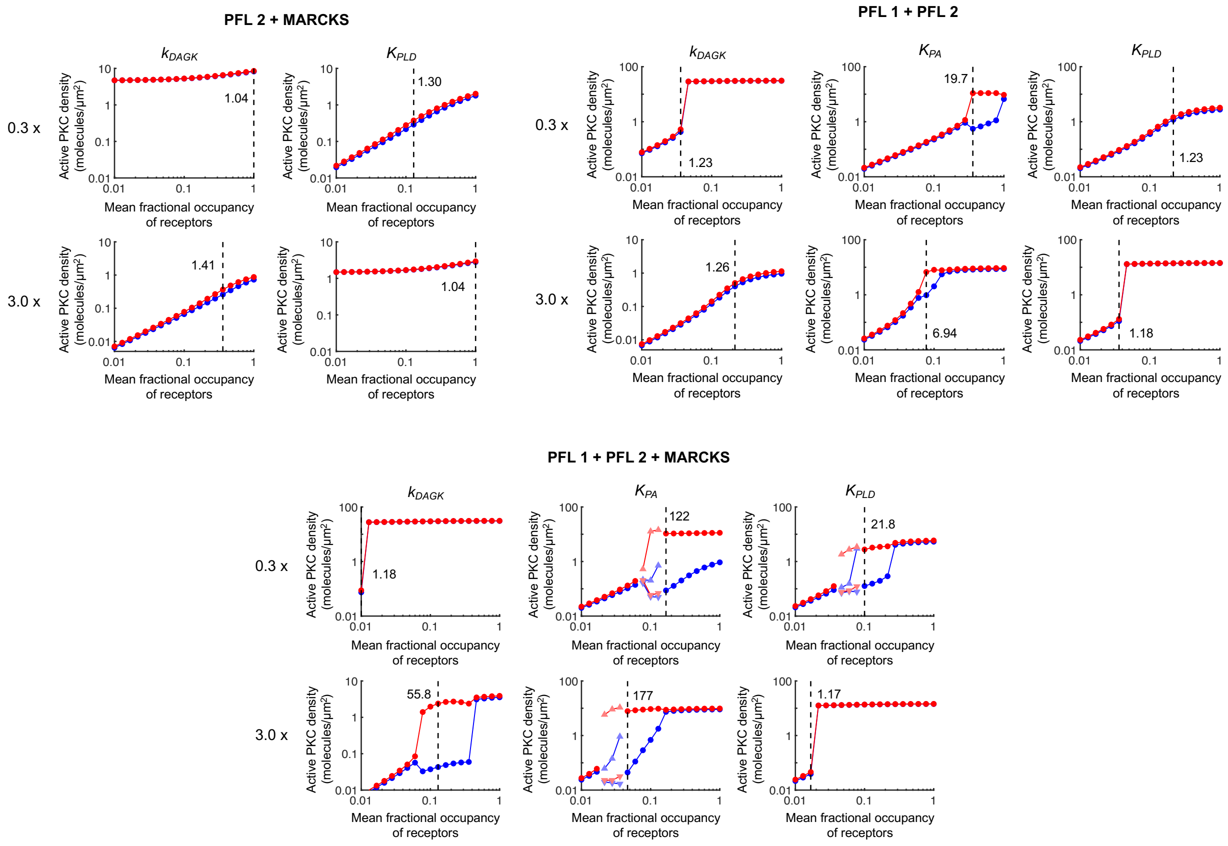

S5 Fig: Variation of key parameters with PFL 2 included in the system. The parameters targeted were k_{DAGK}, which affects the conversion of DAG to PA; K_{PA}, which affects the PA-PLC affinity in PFL 1; and K_{PLD}, which affects the sensitivity of PFL 2 to active PKC. Each parameter was decreased to 0.3X and increased to 3X. Each plot shows active PKC density vs. *rfrac* for 10% gradient steepness, as in **Fig. 6**.
