# Supplementary material for: Mechanistic models of PLC/PKC signaling implicate phosphatidic acid as a key amplifier of chemotactic gradient sensing
Source: PLoS Comput Biol. 2020 Apr 7;16(4):e1007708. doi: 10.1371/journal.pcbi.1007708 (PMC7164671; doi:10.1371/journal.pcbi.1007708)
Supplement: S6 Fig — For each cell i in the simulation, its mean receptor activation ri and the difference in receptor activation across its length Δri were calculated. Cells with receptor activation states above the curve engaged in chemotaxis, whereas those with states below the curve engaged in random migration only. (PDF) [file pcbi.1007708.s008.pdf]

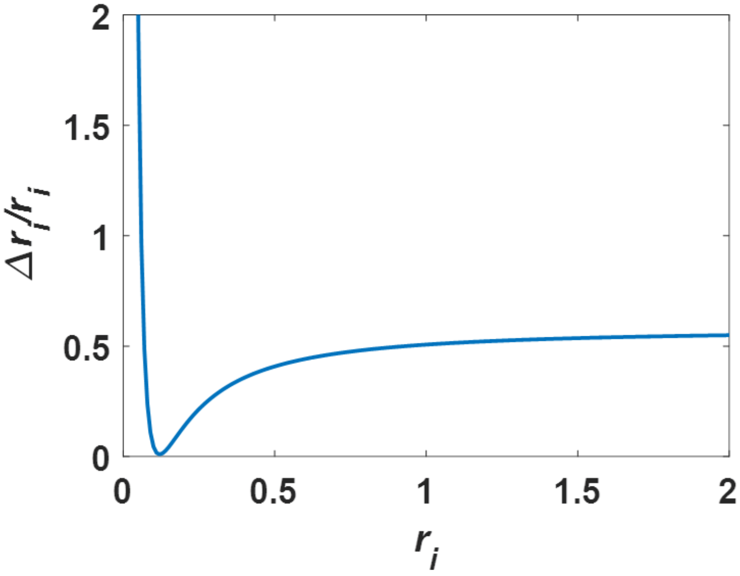

S6 Fig: Plot of Equation 4. For each cell i in the simulation, its mean receptor activation r_i and the difference in receptor activation across its length Δr_i were calculated. Cells with receptor activation states above the curve engaged in chemotaxis, whereas those with states below the curve engaged in random migration only.
