# Supplementary material for: Mechanistic models of PLC/PKC signaling implicate phosphatidic acid as a key amplifier of chemotactic gradient sensing
Source: PLoS Comput Biol. 2020 Apr 7;16(4):e1007708. doi: 10.1371/journal.pcbi.1007708 (PMC7164671; doi:10.1371/journal.pcbi.1007708)
Supplement: Attachment — Submitted filename: review.docx [file pcbi.1007708.s010.docx]

J.L. Nosbisch et al. have developed a mathematical model of PLC/PKC signaling that polarizes in response to shallow chemotactic gradients below 10%. This was achieved by, building on a previous work, including two novel positive feedback loops (PFLs) in the model based on the published data. The first PFL, the PA-mediated stabilization of PLC$\gamma1$ association with the membrane, is modeled as an increased lifetime of the receptor- PLC$\gamma1$ complex at the membrane due to PA. The second PFL, the effect of active PKC on the activity of PLD1, is considered as a source term for PA with a rate that is modeled as a Hill function of active PKC density. Through a rigorous analysis, the authors have shown the effects of turning ‘on’ and ‘off’ of different PFLs on the polarization of the pathway, and have quantified the degrees of responsiveness and robustness of the gradient sensing machinery. They have identified DAG kinases activity as a key parameter influencing the responsiveness of the gradient sensing in the pathway. They have also tested the effects of asymmetry in cell shape geometry on the polarization of the PLC/PKC pathway. And finally, using model-predicted polarization thresholds, they have built and tested a hybrid model of wound healing that yields effective collective migration and predicts the existence of chemotactic wavelets with smaller steepness within the plateau region of the PDFG concentration profile.

The modeling assumptions and simplifications are reasonable, the quantification and representation of the results are intuitive, and the flow of information in the text is very easy to follow. The computational tools used for the numerical simulations are appropriate, and the models/code are available for the readers. Authors have done a great job in executing the numerical investigation of their model, including parameter scanning, step by step analysis of different PFLs, the steady-state analysis, and have provided detailed discussions of the results, model limitations and testable hypotheses based on the models.

I only have a few comments/suggestions.

- in the reaction diagram in Figure 1A, three of the reaction arrows have a different width (thickness of the arrow). If this represents a feature of the diagram that needs to be highlighted it should be explained in the figure caption.

- the effect of cell shape geometry on pathway polarization is investigated by simulating the model inside a static but asymmetric cell. Alternatively, the full shape-dependent problem should be simulated (although I do not believe this is necessary for this manuscript to be an acceptable contribution to Plos Comp Biol), which was suggested by the author as a future model improvement. I think it would be very helpful for the readers to know what mechanistically polarizes the pathway in this model under the conditions of asymmetric shape and uniform PDGF.

- in Figure 3C, the rfrac values corresponding to the max. front/back PKC ratios are missing. I was wondering whether the mean receptor occupancy values were shifted as a result of the shape asymmetry.

- the self-locking of the polarized pathway is an interesting spatial phenomenon, and irrespective of the fact that self-locking could be advantages or disadvantageous for the cell, a more mechanistic explanation for the emergence of irreversibility in the pathway could help readers understand the model better or design future models of the pathway. For example, almost all polarized simulations presented in Figures 4 are irreversible, in contrast with the results in Figure 2, why?
