# Supplementary material for: Mechanistic models of PLC/PKC signaling implicate phosphatidic acid as a key amplifier of chemotactic gradient sensing
Source: PLoS Comput Biol. 2020 Apr 7;16(4):e1007708. doi: 10.1371/journal.pcbi.1007708 (PMC7164671; doi:10.1371/journal.pcbi.1007708)
Supplement: Attachment — Submitted filename: Comments to the Authors_PLOSCB_JN_2019_JH.docx [file pcbi.1007708.s011.docx]

**Author response to Reviewers’ comments on original manuscript # PCOMPBIOL-D-19-01266: "Mechanistic Models of PLC/PKC Signaling Implicate Phosphatidic Acid as a Key Amplifier of Chemotactic Gradient Sensing."**

**We sincerely thank the Reviewers for their careful evaluation and constructive criticisms of our manuscript. Below we respond to all Reviewer comments (copied in *italics* below) and outline the corresponding additions and changes to the manuscript. In the marked version of the manuscript (included as a supplementary file), additions are marked in blue, and all edits are marked by a vertical line in the left margin.**

**Reviewer #1:**

*The authors present an expanded version of a model published previously. This version explores the role of two additional positive feedback loops not considered in the old model. The model analysis and simulations generated several interesting predictions that can be experimentally validated.

Major comments:
1- In the present form, the model is not validated, as the authors did not perform any comparison of model simulations with experimental data. I understand that ideal experimental data to validate the model may not be available, but the authors should, at least, compare PIP2, DAG and PA steady state levels in resting conditions (without chemotactic gradient) with available estimates (even if in different cells) to check that the model is working around a feasible range of concentrations.*

**Response:**

We are confident about certain estimates and less so about others. Our estimates of the total, free, and MARCKS-bound PIP_2_ levels are based on insights from the literature and are the same as in our previous paper (Mohan et al., 2017). Regarding DAG and PA after stimulation, the apparent consensus is that those lipid second messengers are at much lower densities than PIP_2_, and our parameter values reflect that notion. Spurred by the Reviewer’s comment, we performed a literature search and found early radioisotopic labeling studies (e.g., PMIDs 3936489, 2826485); however, no consistent values emerged (even within the same paper, using different labels). In the literature at least, modern lipidomics has not yet emerged to the level of protein analytical methods in quantifying abundance of the relevant lipids in cells.

To address this comment a different way, we have added a new analysis in which we made targeted changes to 5 parameters. As explained in the revised manuscript, proportional changes to three of these parameters increase steady-state DAG and PA levels while maintaining the PA/DAG ratio, while the other two changes attempt to normalize the effect of PA on PLC recruitment via PFL 1 and the recruitment of PKC by DAG. Thus, we showed that polarization is reasonably robust for increases in DAG and PA greater than one log (Fig. 5E). We think this adds nicely to the analysis of PA/DAG ratio from the original manuscript (current Fig. 5A-D).

*2- The authors should also add a sensitivity analysis, so that we can have an idea of the influence of parameter values in model behavior. The authors have studied the impact of some parameter changes that are more relevant for the studied PFLs, but an overall sensitivity analysis would allow us to identify parameters that should be estimated with higher precision or detect problems of identifiability. I suggest the authors should compute the relative sensitivity of "Maximum front/back PKC activity ratio" and "dose-response robustness" to small relative changes in each parameter value.*

**Response:**

To address this, we extended the same modulations of the *k_DAGK_* parameter (0.3X and 3X relative to base-case) to the other adjustable rate parameters in the absence of PFL 2. This analysis, presented in new Supplementary Fig. S4 and described in the revised manuscript, shows that polarization is robust to a 3-fold change of each parameter in at least one direction. And, we were able to explain the scenarios where polarization is broken, as previously shown for 0.3X *k_DAGK_*.

We realize that this is not exactly the traditional sensitivity analysis that the Reviewer suggested, in which small relative changes in each parameter value are made; however, the analysis we did with larger relative changes is both systematic and logical in the context of the discussion of parameters associated with Fig. 5.

We note that two plots from the original version, which explored modulation of the *K_PA_* parameter, were omitted from the current Fig. 5. We considered this information redundant in light of the new analyses described above.

*3- The authors have studied a) PFL1 parameter effects and b) geometric effects in the absence of PFL2. When they study PFL2, they identify some parameter conditions where PFL2 does (or does not) contribute to PKC activity polarization. It would be nice to check (even if in supplementary materials only) if the observations made in the absence of PFL2 (both for a) and b) effects) are also seen in both PFL2 scenarios (PFL2 parameters that influence or not PKC activity polarization). If the observations are different, the differences can be a hint to identify the presence and type of PFL2 in a particular cellular system.*

**Response:**

We have performed the suggested analyses and included some of the results as Supplementary Material. Specifically, we tested the effect of varying key parameters and found there again that PFL 2 is more fragile than PFL 1 (new Fig. S5). We also looked at the geometry effects and confirmed that the system polarizes in the expected way (away from the blunted end) when MARCKS is included in the system. In the case of PFL 1 + PFL 2 (no MARCKS), we expected no geometry-induced polarization but found instead a single condition where polarization occurred but towards the blunted end. Since we consider it beyond the scope of what are trying to say about PFL 2, and we do not yet have a good explanation of the PFL 1 + PFL 2 simulation, we include those results below for the Reviewers only.

*4- PA is also known to activate PI4P5K (*[*https://onlinelibrary.wiley.com/doi/full/10.1002/jcb.21027*](https://onlinelibrary.wiley.com/doi/full/10.1002/jcb.21027)*), an enzyme that produces PIP2. So, PA may activate PIP2 synthesis rate. How would this regulatory interaction interfere with the remaining feedback loops in your system?*

**Response:**

This is a great suggestion that we have now included in the Discussion section under model limitations. As we discuss, our expectation is that this interaction will not interfere with the remaining PFLs; rather, it is fully expected to synergize, since it is coherent with PFL 1 (we have performed some preliminary simulations, with PA enhancing the rate of PIP_2_ synthesis in our model, and those simulations support our hypothesis).

*Minor comments:
1- The meaning of the asterisk (identifying irreversibility of the PKC polarization) should be mentioned in all the figure legends where it appears, and not only in the main text.*

**Response:** Done.

*2- Is it reasonable to have the same diffusivity for DAG and DAG-PKC?*

**Response:**

In theory, the part of the molecule or complex that is inserted in the membrane (the acyl chains) is the primary determinant of mobility. Even a large protein being dragged through the relatively low-viscosity cytoplasm is not expected to slow the mobility much. That said, there could be as-yet-unknown complexities affecting the mobility of a particular complex.

*3- In figure 3C, in the second column, some lines have two numbers (1/16.7). Explain the meaning of the two numbers in the figure legend.*

That table is now in current Fig. 4D. The caption now includes, ‘For the front-end blunted simulations where the ratio is less than 1 (internal gradient opposite the external gradient), the inverse of the ratio is indicated.’

**——————————————————————————————————————————**

**Reviewer #2:**

*In this manuscript, the authors build on two previous model (Refs 17 and 29) to investigate the role of two new feedback loops associated with phosphatidic acid. They show that components of this feedback loops are important regulators of the model and can enhance the robustness of DAG/active PKC polarization. Using this network in a model of wound-healing, they show that efficient collective migration can be achieved with these polarization thresholds. This is an important area of research but the execution of the modeling was lacking.

Major comments (these comments are in the order of my reading the text):
1) Originality: It is very hard to distinguish the substantial new novel contributions from this model as compared to Mohan et al. 2017 [ref 17]. In fact, Figure 1A in very similar in both papers except for the addition of the red reactions in the current manuscript. It is not immediately obvious that the addition of the two new feedback loops as modeled by the reactions in red is a significant advance for publication in PLoS Comp Biol. For a reader to understand this manuscript requires a thorough reading of ref 17 making it hard to be a standalone publication.*

**Response:**

The two new positive feedback loops we consider adds to the previously described regulation of MARCKS for a total of three feedback loops supported by literature. The new feedback loops focus on phosphatidic acid as a lipid second messenger, a species that was not explicitly included in the previous model. This is stated more explicitly now in the Introduction.

Regarding the concern that certain details of Mohan et al. need to be understood before understanding the present manuscript, our approach to the original manuscript was to avoid redundancy and focus on the many new aspects. That said, we acknowledge that some redundancy would be helpful for readers’ understanding. To remedy this, we have included a new section under Results titled ‘Analysis of the mechanisms driving PLC/PKC polarization.’ There are two parts to this section, and the first summarizes the relevant conclusions from Mohan et al. and corroborates them in the context of the present study (new Fig. 3A&B).

*2) There are many abbreviations and assumptions throughout the text limiting accessibility. For example, in Results paragraph 1, the reasoning underlying how PA affects PLC activity were modeled are really assumptions of the model that must be carefully stated in a separate section. Additionally, these assumptions must be revisited in the limitations section of the discussion.*

**Response:**

The nature of the assumptions are spelled out (at different levels) in four different places. First, in the Introduction, we cite the evidence for PFLs 1 & 2. Second, at the beginning of the Results, we spell out in words how the PFLs were described mathematically. This information is then explained in detail under Materials and Methods and Supplementary Text S1. To provide better connectivity, we now explicitly refer to the Materials and Methods and Supplementary Text S1 in the first Results section. We also now remind the reader, in the Discussion section under model limitations, that the mechanisms we consider are incompletely understood.

*The large number of abbreviations and notational issues also make the manuscript hard to follow. For example, using 'e' for active PLC enzyme is a weird notation. For the longest time, I interpreted to be Euler's number and struggled to follow the terms in the supplementary table. A list of abbreviations would also be helpful, although I'm sure the two feedback loops can be named better than PFL1 and PFL2.*

**Response:**

We have gone through the manuscript and taken care to follow the journal’s policy on abbreviations. Thus, we were able to reduce the total number of them. We did keep positive feedback loop (PFL) as an abbreviation, given its frequency of use and association with PFL 1 and PFL 2 mechanisms; in the revised manuscript, we define it at the first mention.

Regarding the notation, those choices (*e*, for example) were largely constrained to be consistent with our previous publication.

*3) Figure 1B: Where is the gradient of the receptors in the spatial geometry? If it is a 2D spatial model (as shown in Figure 3A), then is the receptor gradient simply the difference of receptor at x = 20 and x = -20 divided by 40? Or is it along the arc length? In which case, x doesn't span -20 to +20 but whatever the arc length corresponding to the locations is. Given that it is a 2D spatial model, and the membrane is only the arc length of the curve, the physicality of the imposed receptor gradient doesn't make sense to me.*

**Response:**

We have added new Fig. 1B to explain the model geometry and how receptor activation varies linearly from back to front. The choice of linear with respect to *x*, rather than linear along the arc length, is dictated by the notion that *r*(*x*) is determined by an external, not intrinsic, gradient.

*4) Page 7: This conclusion held true across simulations with extensive sweeps of the PFL parameters. Where are these shown?*

**Response:**

These simulations were performed but were/are not shown. To avoid confusion, we have removed that sentence. The new analysis described in the new section, ‘Analysis of the mechanisms driving PLC/PKC polarization’ (Fig. 3C), explains why it is impossible for PFL 1 to polarize signaling on its own.

*5) Figure 2: The authors should consider using different symbol shapes and not just colors for front and back (this is a minor point). In panel 2A: do the front and back curves overlap throughout?*

**Response:** Yes, where only the red symbols appear, they overlap.

*What is most interesting is that the region close to rfrac of 0.1 is where many oscillatory solutions seem to lie. However, these oscillations are not fully explored. Does this oscillation translate into a spatial pattern? These regimes should be carefully analyzed for stability features since these oscillations are observed in simulations and the underlying cause identified. The issue of oscillations was completely glossed over in the manuscript. Could perturbations in rfrac result in loss of steady state or the maximum front/back ratio? Could stochasticity be important (likely yes, but how?)? Addressing these questions would complete the study and significantly elevate the manuscript.*

**Response:**

We agree that these are all interesting aspects. In our previous publication, we discussed oscillations, and so we chose to shy away from that and focus on the extent and robustness of the stable polarization patterns. Stochastic effects are mentioned in the Discussion and would be a promising avenue to follow up on; our opinion is that it is beyond the scope of the present study.

*6) Figure 2: Another issue is that membrane density of PKC front to back is compared but what does this mean in a spatial model (see comment regarding Figure 1B, the same issue lies here and throughout the manuscript.).*

**Response:** Addressed through the addition of new Fig. 1B.

*7) Figure 3 and associated results: This the first place where the geometry is shown and one asymmetry is introduced. The logic for introducing this asymmetry is not clear. But what becomes clear immediately is that shape effects are important. However, this effect is also not fully explored. Given the 2D model (1D membrane) and the role of shape in signaling (see the many references in ref 46, the authors just gloss over this important issue and move on to other things. This significantly diminishes the potential impact of the work.*

**Response:**

The base geometry is now shown in Fig. 1B. The rationale for the asymmetric geometry presented in current Fig. 4 is now clarified by the aforementioned inclusion of current Fig. 3A, which confirms the requirement for maintaining a gradient of phosphorylated MARCKS in the cytosol explored in Mohan et al. This analysis was repeated for the asymmetric geometry (Fig. 4C) to confirm consistency.

*Also, the difference between the two columns in figure 3C is not clear.*

**Response:**

We have changed this (current Fig. 4) for better clarity and consistency with Fig. 1B&C. In the current version, the external gradient is always from left to right; with respect to the gradient, the left and right ends are always the back and front, respectively.

*8) Figure 4 and 5 and associated results: The authors identify a series of kinetic parameters that are important in determining the ratio of PKC front/back activity. Then they vary these parameters and show that they are important. This is a circular argument. These parameters are important because of the way the model was constructed. Additionally, it is not clear under what physiological conditions these parameters can be measured or manipulated in experiments. Varying copy number of enzymes is a much more meaningful experimental correlate.*

**Response:**

We disagree with the Reviewer’s perception of a circular argument and have hence changed our presentation there, also to address different Reviewer concerns. In the current version, we begin that part of the Results with a systematic variation of rate parameters (without PFL 2; Supplementary Fig. S4). We then discuss our steady-state analysis, which is the basis for understanding why certain parameters are important.

Regarding the variation of enzyme copy number, we are in total agreement and note that the modulation of DAGK activity is discussed along those lines in the Discussion.

*What are the *s in Figure 4? They are not defined in the caption of this or previous figures.*

**Response:** Fixed as described in response to Reviewer 1.

*9) Sensitivity analysis: sensitivity analysis of the kinetic parameters and initial conditions should be included, particularly in light of the oscillatory regimes. What if the diffusion constants were altered? Then the results across the entire manuscript would change I would think? It would be important to report to what extent the model is robust to these changes. Plus the diffusion constants must be reported from literature where available.*

**Response:**

Addressed above with newly added Fig. 3A&B and Supplementary Fig. S4 and associated manuscript text.

*10) If receptors were allowed to diffuse freely rather than maintain an active gradient across the membrane (after correcting for the geometry issues raised above) would a steady state receptor gradient exist? That seems unlikely given that studies have shown that receptors diffuse in the plane of the membrane and diffusion serves to dissipate the gradient. This should be explored a carefully because this gradient is the input to the model.*

**Response:**

Most transmembrane proteins show highly restricted mobility, with D ~ 0.01 μm^2^/s serving as a conservatively high order-of-magnitude estimate (FRAP experiments have suggested far lower). With an also conservatively high estimated lifetime of ~ 10 minutes (limited by endocytosis if not by ligand dissociation), the estimated maximum spatial range is ~ 2.5 μm, an order of magnitude below cellular dimensions.

*11) Testable hypotheses: Regarding the experiments proposed by the authors to test molecular mechanisms, it is not as trivial as they propose to use isoform selective inhibitors. First, the model predicts that kinetic parameters are important, which are near impossible to alter in situ. Interestingly, this section has the least references to plausible experimental modalities, so it is not clear if these methods are even possible.*

**Response:**

The perturbations that we primarily draw attention to are PKC inhibition (standard) and modulation of DAG kinase activity. We acknowledge that the latter is not standard. As clarified in multiple places now, the DAG kinase parameter is a lumped parameter that would be modulated by both DAG kinase enzymatic activity and DAG kinase expression/abundance. Both avenues are mentioned in the Discussion. Although we are wary of the available DAG kinase inhibitors, we now cite two relevant references.

*12) References: the fraction of self-citation is rather high. I appreciate that the authors have done a substantial amount of work in this field, but they have also missed many fundamental citations in cell shape regulation of signaling (see comment about the references in 46) and receptor diffusion.*

**Response:**

We appreciate this point and have removed two non-essential self-citations and have added citations to two more recent papers related to cell shape regulation of signaling.

**———————–––––––––––––––––––––––––––––––––––––––––––––––––––––––––––––––**

**Reviewer #3:**

*J.L. Nosbisch et al. have developed a mathematical model of PLC/PKC signaling that polarizes in response to shallow chemotactic gradients below 10%. This was achieved by, building on a previous work, including two novel positive feedback loops (PFLs) in the model based on the published data. The first PFL, the PA-mediated stabilization of PLC*$\gamma1$ *association with the membrane, is modeled as an increased lifetime of the receptor- PLC*$\gamma1$ *complex at the membrane due to PA. The second PFL, the effect of active PKC on the activity of PLD1, is considered as a source term for PA with a rate that is modeled as a Hill function of active PKC density. Through a rigorous analysis, the authors have shown the effects of turning ‘on’ and ‘off’ of different PFLs on the polarization of the pathway and have quantified the degrees of responsiveness and robustness of the gradient sensing machinery. They have identified DAG kinases activity as a key parameter influencing the responsiveness of the gradient sensing in the pathway. They have also tested the effects of asymmetry in cell shape geometry on the polarization of the PLC/PKC pathway. And finally, using model-predicted polarization thresholds, they have built and tested a hybrid model of wound healing that yields effective collective migration and predicts the existence of chemotactic wavelets with smaller steepness within the plateau region of the PDFG concentration profile.*

*The modeling assumptions and simplifications are reasonable, the quantification and representation of the results are intuitive, and the flow of information in the text is very easy to follow. The computational tools used for the numerical simulations are appropriate, and the models/code are available for the readers. Authors have done a great job in executing the numerical investigation of their model, including parameter scanning, step by step analysis of different PFLs, the steady-state analysis, and have provided detailed discussions of the results, model limitations and testable hypotheses based on the models.*

*I only have a few comments/suggestions.*

*1- In the reaction diagram in Figure 1A, three of the reaction arrows have a different width (thickness of the arrow). If this represents a feature of the diagram that needs to be highlighted it should be explained in the figure caption.*

**Response:** Fixed (arrows are now similar thickness).

*2- The effect of cell shape geometry on pathway polarization is investigated by simulating the model inside a static but asymmetric cell. Alternatively, the full shape-dependent problem should be simulated (although I do not believe this is necessary for this manuscript to be an acceptable contribution to PLoS Comp Biol), which was suggested by the author as a future model improvement. I think it would be very helpful for the readers to know what mechanistically polarizes the pathway in this model under the conditions of asymmetric shape and uniform PDGF.*

**Response:**

This was addressed in response to other Reviewers. Briefly, the geometry effect is attributed to formation of a gradient of phosphorylated MARCKS in the cytosol. This is set up in the new section associate with new Fig. 3.

*3- In Figure 3C, the rfrac values corresponding to the max. front/back PKC ratios are missing. I was wondering whether the mean receptor occupancy values were shifted as a result of the shape asymmetry.*

**Response:**

No, the maximum front/back active PKC ratios are occurring at *rfrac* = 0.10 for all but one of the scenarios listed in the chart (current Fig. 4D). In that case, it occurred at *rfrac* = 0.13.

*4- The self-locking of the polarized pathway is an interesting spatial phenomenon, and irrespective of the fact that self-locking could be advantages or disadvantageous for the cell, a more mechanistic explanation for the emergence of irreversibility in the pathway could help readers understand the model better or design future models of the pathway. For example, almost all polarized simulations presented in Figures 4 are irreversible, in contrast with the results in Figure 2, why?*

**Response:**

We have attempted to address this but honestly don’t have a great answer. The question is difficult, because concepts such as bistability that might explain irreversibility are intractable for spatially extended systems, except when certain conditions apply (PMID 24158538). Such a simplification can’t be applied to our system because of the important phospho-MARCKS and PIP_2_ gradients in the cytosol and membrane, respectively (their diffusion cannot be considered either very slow or very fast).

One of our attempts to answer this question is the *e*- and *d*-nullcline analysis shown in new Fig. 3C. While we think this analysis is useful in explaining polarization, it does not adequately predict reversibility versus irreversibility.
